# Applying Science: Opportunities to Inform Disease Management Policy with Cooperative Research within a One Health Framework

**DOI:** 10.3389/fpubh.2015.00276

**Published:** 2016-01-08

**Authors:** Jason K. Blackburn, Ian T. Kracalik, Jeanne Marie Fair

**Affiliations:** ^1^Spatial Epidemiology and Ecology Research Laboratory, Department of Geography, University of Florida, Gainesville, FL, USA; ^2^Emerging Pathogens Institute, University of Florida, Gainesville, FL, USA; ^3^Cooperative Biological Engagement Program, Defense Threat Reduction Agency, Fort Belvoir, VA, USA

**Keywords:** disease surveillance, disease modeling, anthrax, brucellosis, plague, tularemia, one health

## Abstract

The ongoing Ebola outbreak in West Africa and the current saiga antelope die off in Kazakhstan each represent very real and difficult to manage public or veterinary health crises. They also illustrate the importance of stable and funded surveillance and sound policy for intervention or disease control. While these two events highlight extreme cases of infectious disease (Ebola) or (possible) environmental exposure (saiga), diseases such as anthrax, brucellosis, tularemia, and plague are all zoonoses that pose risks and present surveillance challenges at the wildlife-livestock–human interfaces. These four diseases are also considered important actors in the threat of biological terror activities and have a long history as legacy biowarfare pathogens. This paper reviews recent studies done cooperatively between American and institutions within nations of the Former Soviet Union (FSU) focused on spatiotemporal, epidemiological, and ecological patterns of these four zoonoses. We examine recent studies and discuss the possible ways in which techniques, including ecological niche modeling, disease risk modeling, and spatiotemporal cluster analysis, can inform disease surveillance, control efforts, and impact policy. Our focus is to posit ways to apply science to disease management policy and actual management or mitigation practices. Across these examples, we illustrate the value of cooperative studies that bring together modern geospatial and epidemiological analyses to improve our understanding of the distribution of pathogens and diseases in livestock, wildlife, and humans. For example, ecological niche modeling can provide national level maps of pathogen distributions for surveillance planning, while space-time models can identify the timing and location of significant outbreak events for defining active control strategies. We advocate for the need to bring the results and the researchers from cooperative studies into the meeting rooms where policy is negotiated and use these results to inform future disease surveillance and control or eradication campaigns.

## Introduction

The recent Ebola outbreak in West Africa (1, 2) has been a shocking reminder of the ever present risk of rapidly spreading disease outbreaks and the reality of the difficulties involved in outbreak response (3) and surveillance. That outbreak, resulting in more than 10,000 human deaths (and still ongoing at the time of this writing), highlights the severity that re-emerging diseases can pose. The difficulties in identifying the potential zoonotic source of this infection (4) highlight the importance of understanding interactions at the human–wildlife interface (5) and of the importance of wildlife surveillance. The ongoing saiga antelope, *Saiga tatarica*) die off in Kazakhstan, where more than 100,000 antelope have died in <8 weeks^1^ (representing approximately 1/2 of all remaining saiga), presents another example of a severe loss of animals to unknown environmental contamination or pathogen exposure. While the ongoing Ebola outbreak and saiga die off represents the extreme of outbreak consequences (high human or wildlife mortality), several other important zoonoses have been re-emerging or maintaining with high incidence in known endemic areas. Diseases such as anthrax, brucellosis, tularemia, and plague are all zoonoses that pose risks and present surveillance challenges at the wildlife-livestock–human interfaces. These four diseases are also considered the most important pathogens for use in biological terror activities and have a long history as legacy biowarfare pathogens. Inter-specific transmission of each of these diseases demands that surveillance should include coordination between veterinary and human health personnel. This paper will review recent studies done cooperatively between American and institutions within nations of the Former Soviet Union (FSU) focused on spatiotemporal, epidemiological, and ecological patterns of these four zoonoses. These cooperative studies were all funded by the Defense Threat Reduction Agency’s Cooperative Biological Engagement Program (or previous iterations of the program such as the Biological Threat Reduction Program). The goal of those efforts was to bring local and American scientists together to apply contemporary geospatial and epidemiological techniques to the issues of zoonotic disease persistence and transmission in the FSU with an emphasis on pathogens on the US Federal Select Agent Program pathogen list^2^.

These projects were designed to be aimed at understanding disease spatiotemporal patterns and ecology. While the disease systems, study sites, and geospatial techniques differed, each of the studies highlighted here are related in that they applied geospatial modeling techniques to better understand disease patterns in humans, livestock, or wildlife. From these examples, we identify how study results may be use to inform national and international policy making to improve disease surveillance and inform control strategies. Broadly, these techniques rely on surveillance-derived datasets describing either case reports or serological evidence of disease presence at a spatial resolution smaller than the national level; most data were either farm or village locations mapped as GPS coordinate pairs or data aggregated to the rayon (district equivalent). Across the studies that we survey in this paper, it is noteworthy to point out a significant DTRA investment in data development for these projects in terms of data access, data compilation, and personnel time required. Such efforts are instrumental in cooperative biological engagements and are an important and often undervalued part of disease ecology studies.

This paper illustrates how these techniques can inform disease surveillance, control efforts and impact policy. Our focus is to posit ways to apply science to disease management policy and actual management or mitigation practices. As part of this, we emphasize the importance of data sharing between human and veterinary health professionals and suggest actionable ways of improving data sharing. The objective of this paper were to review four diseases (anthrax, brucellosis, tularemia, and plague), that are important to cooperative biological engagement programs, in relation to published research studies and the resulting impact on policy and management decisions.

## Pathogens and Policies

### Anthrax

Globally, anthrax is an important zoonosis with rapid onset and high mortality in wildlife and livestock, a cause of secondary human cases, and a security risk as a bioterror agent (6). The disease, caused by the Gram-positive, spore-forming bacterium *Bacillus anthracis*, can exert significant impacts on wildlife and livestock populations (7, 8). Human cases are most commonly associated with slaughtering infected animals (9). However, anthrax ecology and transmission remain poorly understood and understudied (10). Broadly, outbreaks in enzootic regions arise in specific environmental conditions (11), such as semi-arid grasslands and steppes, and at times of seasonal transitions in climate (12–14). This is true for livestock and wildlife populations. Reports of wildlife cases are becoming more common in North America. Wildlife outbreaks are regular in white-tailed deer, *Odocoileus virginianus*, in Texas (10) and bison, *Bison bison* ssp., in Canada (15). Globally, livestock outbreaks have also been associated with contaminated animal feed (16).

Anthrax occurs nearly worldwide with the heaviest livestock and human disease burden in resource limited countries (17). Animal cases occur across much of the globe, but human cases are most concentrated in Central Asia, Southern Africa, and the Caucasus. Over the last two decades, based on reviews from Promed Mail, Kyrgyzstan had some of the highest reported human case numbers. Additionally, Turkey (18) and Georgia (19) have both reported high numbers of human cases; Georgia has seen significant increases in human anthrax in the last few years (20). Disease control in these areas requires approaches that provide livestock control, such as vaccination campaigns and increased animal surveillance (21) and education programs targeting animal handlers and local slaughter operations.

### Anthrax in Azerbaijan and Georgia

A recent cooperative study by Kracalik et al. (22) examined the spatial patterns of human anthrax in Azerbaijan looking at three time periods: Soviet (1984–1991), post-Soviet (1992–1995), and post-independence following livestock vaccination campaigns (1996–2010). Generally, the rate of human anthrax increased from the Soviet to the post-Soviet periods, which was most likely due to the drastic decrease in funding and changes in livestock management associated with Azerbaijani independence. From the post-Soviet through the post-independence period, there was a drastic decrease in the overall human incidence rate and a geographic shift in the concentration of reporting. These results suggest that livestock-associated human anthrax can be controlled with livestock vaccination campaigns. Furthermore, these findings indicate that surveillance cannot focus solely on areas of historic disease presence, but rather needs to be dynamic and sensitive to changes in livestock distributions and socioeconomic shifts associated with agricultural production.

Like Azerbaijan, Georgia has seen an increase in human anthrax cases over the last decade, characterized by a drastic increase over the past 5 years (20). In recent years, Georgia has seen some of the highest rates of human anthrax globally. Cooperative research has shown the during the period 2000–2009, clusters of human anthrax cases in eastern and west central Georgia were associated ecological conditions that promote pathogen persistence (19). Consistent with research in Azerbaijan, these studies identified spatial heterogeneity across the Georgia landscape suggesting control efforts should be targeted to prioritize high risk areas. One major contrast between the two countries is the change in livestock vaccination policy, while Azerbaijan maintained vaccination through the last decade, Georgia ended compulsory vaccination in 2007, which lead to a drastic decrease in the number of animals vaccinated. This compulsory policy was nominally reinstated in 2012 following a historical rise in cases (20), though the total coverage of animals remains low. In both countries, anthrax surveillance remains highly anthropocentric, making it difficult to identify livestock populations at greatest risk. The use of spatial analyses in these studies provides a starting point for identifying areas where livestock surveillance should be prioritized.

Another important finding of the cooperative research in Georgia is related to the human populations at risk. Classically, anthrax is considered a rural disease. However, the recent increase in Georgian anthrax has been associated with an increase in peri-urban and urban dwellers likely associated with the selling of contaminated meat (19, 20). From a policy perspective, these results indicate a need for increased inspection and regulation of meat markets and sources of meat. Likewise, marketplaces may serve as points of outreach for public health education campaigns directed at both meat consumers and meat producers.

### Anthrax in Kazakhstan

The Central Asian Steppe has a long history of livestock and human anthrax (23). Using data compiled from 1930 to 2006, several cooperative studies have increased our knowledge of the spatiotemporal, and ecological distribution of anthrax in Kazakhstan (21, 24–27). These studies have implemented predictive modeling approaches to map the distribution of anthrax and *B. anthracis* in Kazakhstan. Research by Joyner et al. (26) used livestock anthrax outbreak locations reported between 1960 and 2000 to develop an ecological niche model. A subsequent study refined those predictions to identify areas of anthrax risk by using a combination of spatial analysis and generalized linear modeling (21). Both the ecological niche and risk models identify areas that can be used to define surveillance areas, with risk models best used to prioritize areas for preemptive annual livestock vaccination campaigns. From a policy perspective, passive surveillance zones require laboratory infrastructure and veterinary training to identify and test for anthrax should spring or summer time livestock die offs present. These studies illustrate specific examples of how maps of pathogens (ecological niche models) or disease risk (predicting clusters) can be used to prioritize surveillance and control. Such approaches can be introduced into other countries where anthrax surveillance and control remains a challenge.

### Anthrax in Ukraine

Like Kazakhstan, Ukraine has a long documented history of livestock and human anthrax (28). Two cooperative studies have recently been published that illustrate the changing distribution of livestock anthrax in Ukraine. First, Bezymennyi et al. (28) examined spatial patterns of livestock anthrax (1913–2012) in pre- and post-Soviet Ukraine. Like many countries, during the last 50 years, Ukraine saw a drastic reduction in the overall reports of anthrax and a contraction of the geographic area where anthrax persists. Following the dissolution of the Soviet Union, livestock anthrax increased briefly (1992–1997). During the last 17-years, there has been a reduction in reporting and a contraction of anthrax foci on the landscape, although compulsory vaccination in Ukraine extends only to state owned livestock operations. Independent livestock owners are less likely to vaccinate, as they bear the burden of cost. A recent outbreak in eastern Ukraine illustrated the ongoing risk when an unvaccinated cow died of anthrax, and was subsequently fed to a domestic dog that also succumbed to infection (29). Furthermore, there was an attempt to sell the infected meat at a market, which could have resulted in human cases had the public health service not halted its sale (28). From a policy perspective, these studies identify the need to examine definitions of compulsory vaccination and the cost benefits of requiring private owners to bear vaccination costs. Similar to the situation in Georgia, these studies also identify the need to evaluate meat inspection processes and to meet regulatory infrastructure to better identify potential sources of contamination meat being sold to the public.

An additional published collaborative study highlights the importance of including wildlife and surveillance for zoonotic diseases (30). In that study, the serological survey of wild boar collected from hunter check stations confirmed that at least some boars are exposed to *B. anthracis* in Ukraine. Most interesting was the fact that positive boars were identified in proximity to historical hotspots of anthrax but not directly overlapping with them. From a policy perspective, these results suggest surveillance should not be limited to livestock and should include wildlife populations. A similar strategy could be employed in Kazakhstan where there is potential overlap between saiga antelope and livestock (31) and may also be useful in the Caucasus, particularly Georgia, where livestock anthrax has been increasing.

### Brucellosis

Brucellosis is one of the most widespread zoonotic diseases worldwide and is regarded as an emerging and re-emerging threat to public and veterinary health worldwide (32). Controlling brucellosis in humans is dependent upon limiting or reducing infection in livestock. Despite the availability of effective livestock vaccines for *Brucella spp*., the disease continues to pose a global public health threat. Regions most heavily burdened by the disease include countries of the Mediterranean, Central Asia, Middle East, Latin America, Sub-Saharan African, and Balkan Peninsula (33). The causative agents of the disease are a group of pathogenic bacteria in the genus *Brucella*, which primarily infect animal reservoirs. Humans are often secondarily infected through the consumption of unpasteurized dairy products or coming into contact with infected animals during animal husbandry or meat processing (32). The primary agents of infection in humans are *Brucella abortus* (cattle), *Brucella melitensis* (sheep and goats), *Brucella suis* (swine), and *Brucella canis* (dogs) (34).

### Brucellosis in Azerbaijan

The disease was first reported from Azerbaijan in 1922 and quickly spread to more than two-thirds of the rayons in <30 years (35). Recent governmental changes brought on by the collapse of the Soviet Union have likely contributed the persistence of the disease, due primarily to decreased funding for surveillance and eradication programs (33). A recent cooperative study evaluated changes in the distribution of human brucellosis over each of three 5-year intervals from 1995 to 2009 (36). That study documented rayon-level disease persistence in humans that can direct contemporary surveillance efforts. As was suggested for Georgia, these data can be used to evaluate the cost-effectiveness and human health impacts of livestock disease control programs.

### Brucellosis in Georgia

Brucellosis is an endemic livestock disease in Georgia with a relatively high human burden (37). In a recent study, data from a contemporary livestock serological survey were used to estimate true disease prevalence in a Bayesian framework comparing each of three important areas within Georgia (Imereti – west central, Kvemo Kartli – southern, and Kakheti – eastern) (38). In total, these three regions represent approximately 45% of George’s milk production and total livestock population. Results from this study of livestock match a recent study of human brucellosis rates in Georgia (39). From a policy perspective, the results of the livestock surveillance study can be used to inform brucellosis control and eradication campaigns focusing first on the areas with greatest livestock brucellosis prevalence. It is likely that control in these areas would result in immediate and measurable improvements in human brucellosis incidents. As was illustrated in the anthrax work of Kracalik et al. (20, 22) in Azerbaijan, surveillance data from humans and animals could be analyzed in a one health framework that directly measure the impact of livestock control on human disease burden.

### Plague

Plague is a flea borne zoonosis caused by the Gram-negative bacterium *Yersinia pestis* (40). Since the onset of the most recent pandemic, which started in China during the mid-nineteenth century, the geographic range of plague has greatly expanded (40). Classically, *Y. pestis* is maintained a sylvatic transmission cycle between partially resistant rodent hosts and adult hematophagous fleas (40) and foci can be maintained indefinitely in enzootic or maintenance cycles as long as sufficient numbers of rodent hosts and flea vectors are present. Natural plague reservoirs are active in Asia, and parts of the Russian Federation (41). Human plague cases have recently reemerged in this region (42).

### Plague in Azerbaijan

Morris et al. (43) used historical maps of plague hosts derived annually between 1972 and 1985 to identify areas on the Azerbaijani landscape were plague carrying mammal densities were high and stable across years. The study digitized historical maps into active GIS layers and applied modern spatial analyses to evaluate areas of disease stability. The goal of the effort was to identify environmental conditions and geographic areas of historical sampling that may identify priority areas for contemporary sampling. In the years following Soviet independence, funds for plague surveillance were limited creating a gap in surveillance needing to be filled. That study identified a few key areas on the Azerbaijani landscape where plague may reemerge today. Beyond identifying those locations, Morris et al. (43) also identified which of those areas were in closest proximity or directly overlapping with increasing human populations. A similar effort field survey in neighboring Iran went a step further and use serological screening of dogs and small rodents and confirmed that historically defined foci could be active as many as two decades after the most recent zoological surveillance (44). From a policy perspective, the Morris et al. (43) all study in Azerbaijan can be used to prioritize exploratory surveillance in historically defined plague foci using serological testing or PCR-based methods. The areas identified as having increasing human population can further be used to prioritize those areas most important for surveillance. In this example, those areas with historically high rodent populations that saw little development until recently should be considered areas of highest likelihood of overlap between those contemporary rodent populations and human encroachment into those habitats.

### Tularemia

*Francisella tularensis*, the causative agent of tularemia, is a zoonotic, Gram-negative bacterium that is broadly distributed across the Northern Hemisphere (45). Human exposure may occur through various pathways including arthropod bites, ingesting contaminated food products or liquids, inhaling aerosolized bacteria, or handling infected animals (45). Despite a global decline in reported human cases (46), tularemia has recently (re)emerged in several countries [summarized by Hightower et al. (47)] Historically, outbreaks in the FSU were linked to small mammals and arthropods (ticks), possibly related to increases host or vector population abundance or density or water-borne outbreaks. Tularemia foci were previously described in the 1960s across a limited geography in the south of Ukraine where several arthropods and small mammals were recognized as competent vectors and hosts (48; 49). However, contemporary characterizations of the spatial distribution and composition of vectors and hosts remains incomplete and should remain a priority in countries with known tularemia outbreaks (47).

### Tularemia in Ukraine

Tularemia has a long history in Ukraine. In an effort to understand the historical distribution and identify possible areas of contemporary surveillance, Hightower et al. (47) mapped the spatial patterns of historical *F. tularensis* isolates from the Ukrainian Central Sanitation and Epidemiological Station (CSES; now Ukrainian Center for Disease Control) and tested for space-time clusters on a database spanning more than 60 years. That study identified several historical foci that may serve as areas of persistence where disease reemergence is likely in humans. Additionally, that study defined tick vector and mammalian host species that should be priorities for sylvatic surveillance efforts. Hightower (50) use those data to construct small mammal and tick species-specific ecological niche models to estimate the potential geographic distribution of the pathogen across Ukraine. From a policy perspective, these studies provide Ukraine with specific local areas where disease surveillance should be focused. The space-time clusters defined can also serve as a baseline for comparing contemporary surveillance results. Additionally, Hightower et al. (47) identified areas where potential environmental exposure to contaminated crops may serve as an important transmission source that may not be detected from small mammal surveillance. These areas require additional infrastructure for testing such samples in the absence of human cases.

### A Call for One Health Strategies for Improved Disease Surveillance and Control

As illustrated in the examples presented here, these zoonoses cross the human/livestock/wildlife interface. Because of this, effective surveillance and control strategies require a one health approach. These strategies should target different populations (human, livestock, wildlife) across the geography of the diseases. Central Asia and the Caucasus require improved livestock surveillance and vaccination strategies aimed at reducing the livestock burden of disease; this is true for both anthrax and brucellosis. Such strategies should have significant livestock and human health benefits. In contrast, the wildlife situation of anthrax in wild boar in Ukraine poses a different challenge. Anthrax vaccination is untenable in wildlife (11, 51). In the absence of vaccination, rapid carcass cleanup during outbreaks is the only apparent means of reducing the size of outbreaks (51). However, it is important that burial efforts result in deep burial to reduce potential for inadvertent digging that exposes carcasses, as bone can remain infectious for long periods of time. Because of this, there is a need to better understand the timing and spatial distribution of epizootics; such information comes from increased surveillance and environmental sampling. This would allow managers to stage preemptive surveillance and control efforts.

Ecological niche models (also referred to as species distribution models) can be used to broadly define the geographic range of *B. anthracis, Y. pestis*, and *F. tularensis* to better inform surveillance efforts. When coupled with spatial analyses of outbreaks, we can identify areas of high risk (where the clusters occur) and areas where passive surveillance should increase (where niche models predict in under investigated areas). Specific to anthrax, expanded surveillance and wildlife telemetry studies can assist in understanding the relationship between individual animals in a herd and their use of the landscape during anthrax risk periods (11). Such studies can shed light on the role of animal behavior in contacting the environmental reservoir for the pathogen. This could greatly improve our understanding of anthrax in Ukrainian boar populations.

Much of the recent spatial modeling of anthrax has relied on mortality data to understand the disease, which likely underestimates the extent and intensity of the disease (52). The data from boars suggest that pathogen exposure occurs beyond known foci, even without reported mortality events. Coupling data from across temporal and spatial scales and across host species in a modeling framework should provide better information on the disease that can be shared with wildlife managers, regional public health officers, and policy makers.

We have also illustrated that disease surveillance in these countries is anthropocentric and requires greater data sharing, cooperation, and funding to support joint human and veterinary surveillance and disease control. The work on anthrax in Azerbaijan highlights the role of livestock disease control for improving human health. Future efforts should expand this type of research to brucellosis studies across these countries. Regional approaches to zoonotic and transboundary diseases often require regional efforts in mitigation and vaccination strategies that are effective in reducing the propagation of the infectious diseases. Through the above collaborations discussed in this paper, the countries of Azerbaijan, Georgia, Kazakhstan, and Ukraine have developed a Regional Disease Surveillance Working Group (RDSWG) to foster communication and collaboration in disease surveillance of these pathogens. This is in direct response to the cooperative engagements with the countries and the results of research studies that show the importance of sharing of data and communication in reducing the impact of transboundary diseases.

The examples of plague and tularemia presented here illustrate the importance of continued small mammal and associated vector surveys across these countries. Each of these diseases is maintained in small mammal populations and is likely to maintain over long periods of time. Ultimately, surveillance is time-consuming and expensive and must be balanced against risk. In the work in Azerbaijan, Morris et al. (43) illustrated the use of high resolution spatial data mapping human populations can be compared to areas of historical disease foci to focus what are realistically limited surveillance dollars to those areas of greatest likelihood of human infection.

## Conclusion

Across these examples, we have illustrated the value of cooperative studies that bring together modern geospatial and epidemiological analyses with historical and contemporary disease surveillance to improve our understanding of the distribution of pathogens and diseases in livestock, wildlife, and humans. The results of these efforts illustrated in this paper are all available as peer-reviewed studies. We advocate for the need to bring the results and the researchers from cooperative studies into meetings where policy is negotiated to best use these results to inform future disease surveillance and control or eradication campaigns. Each of the studies highlighted here identify local spatial heterogeneity in the distributions of these diseases. Such information should be considered critical for policymakers when considering strategies for reducing eradicating these diseases.

## Conflict of Interest Statement

Dr. Jeanne Fair is currently a program officer with the Defense Threat Reduction Agency. She was not involved with the studies reviewed in this paper, nor involved in this decisions to fund those projects. Jason K. Blackburn and Ian T. Kracalik have no conflict of interest to declare.

## Footnotes

^1^http://www.mnn.com/earth-matters/animals/stories/why-are-saiga-antelope-dying-in-record-numbers

^2^http://www.selectagents.gov/

## References

[1] Frieden TR Damon I Bell BP Kenyon T Nichol S Ebola 2014 – new challenges, new global response and responsibility. N Engl J Med (2014) 371:1177–80. 10.1056/NEJMp1409903 25140858

[2] Dixon MG Schafer IJ Ebola viral disease outbreak – West Africa, 2014. MMWR Morb Mortal Wkly Rep (2014) 63:548–51. 24964881 PMC5779383

[3] Ansumana R Bonwitt J Stenger DA Jacobsen KH Ebola in Sierra Leone: a call for action. Lancet (2014) 384:303 10.1016/S0140-6736(14)61119-3 25002177

[4] Gire SK Goba A Andersen KG Sealfon RS Park DJ Kanneh L Genomic surveillance elucidates Ebola virus origin and transmission during the 2014 outbreak. Science (2014) 345:1369–72. 10.1126/science.1259657 25214632 PMC4431643

[5] Alexander KA Lewis BL Marathe M Eubank S Blackburn JK. Modeling of wildlife-associated zoonoses: applications and caveats. Vector-Borne Zoonotic Dis (2012) 12:1005–18. 10.1089/vbz.2012.0987 23199265 PMC3525896

[6] Blackburn J Integrating geographic information systems and ecological niche modeling into disease ecology: a case study of *Bacillus anthracis* in the United States and Mexico. In: Skowroski EW. O’Connell KP, editors. Emerging and Endemic Pathogens: Advances in Surveillance, Detection, and Identification. Dordrecht: Springer (2010). p. 59–88.

[7] Hugh-Jones M De Vos V. Anthrax and wildlife. Rev Sci Tech (2002) 21:359–83. 11974621 10.20506/rst.21.2.1336

[8] Fasanella A Galante D Garofolo G Jones MH. Anthrax undervalued zoonosis. Vet Microbiol (2010) 140:318–31. 10.1016/j.vetmic.2009.08.016 19747785

[9] Woods CW Ospanov K Myrzabekov A Favorov M Plikaytis B Ashford DA. Risk factors for human anthrax among contacts of anthrax-infected livestock in Kazakhstan. Am J Trop Med Hyg (2004) 71:48–52. 15238688

[10] Blackburn JK Hadfield TL Curtis AJ Hugh-Jones ME Spatial and temporal patterns of anthrax in White-Tailed Deer, *Odocoileus virginianus*, and hematophagous flies in West Texas during the summertime anthrax risk period. Ann Assoc Am Geogr (2014) 104:939–58. 10.1080/00045608.2014.914834

[11] Blackburn JK McNyset KM Curtis A Hugh-Jones ME Modeling the geographic distribution of *Bacillus anthracis*, the causative agent of anthrax disease, for the contiguous United States using predictive ecologic niche modeling. Am J Trop Med Hyg (2007) 77:1103–10. 18165531

[12] Turner A Galvin J Rubira R Miller G. Anthrax explodes in an Australian summer. J Appl Microbiol (1999) 87:196–9. 10.1046/j.1365-2672.1999.00869.x 10475947

[13] Parkinson R Rajic A Jenson C. Investigation of an anthrax outbreak in Alberta in 1999 using a geographic information system. Can Vet J (2003) 44:315–8. 12715984 PMC372251

[14] Blackburn JK Goodin DG Differentiation of springtime vegetation indices associated with summer anthrax epizootics in West Texas, USA deer. J Wildl Dis (2013) 49:699–703. 10.7589/2012-10-253 23778625

[15] Dragon D Elkin B Nishi J Ellsworth T. A review of anthrax in Canada and implications for research on the disease in northern bison. J Appl Microbiol (1999) 87:208–13. 10.1046/j.1365-2672.1999.00872.x 10475950

[16] Fasanella A Garofolo G Hossain M Shamsuddin M Blackburn J Hugh-Jones M Bangladesh anthrax outbreaks are probably caused by contaminated livestock feed. Epidemiol Infect (2012) 1:1–8. 10.1017/S0950268812001227 PMC9151811 22814512

[17] Hugh-Jones M 97 global anthrax report. J Appl Microbiol (1999) 87:189–91. 10.1046/j.1365-2672.1999.00867.x 10475945

[18] Özkurt Z Parlak M Tastan R Dinler U Saglam YS Ozyurek SF Anthrax in Eastern Turkey, 1992–2004. Emerg Infect Dis (2005) 11:1939 10.3201/eid1112.050779 16485484 PMC3367647

[19] Kracalik IT Malania L Tsertsvadze N Manvelyan J Bakanidze L Imnadze P Evidence of local persistence of human anthrax in the country of Georgia associated with environmental and anthropogenic factors. PLoS Negl Trop Dis (2013) 7:e2388. 10.1371/journal.pntd.0002388 24040426 PMC3764226

[20] Kracalik I Malania L Tsertsvadze N Manvelyan J Bakanidze L Imnadze P Human cutaneous anthrax, Georgia 2010–2012. Emerg Infect Dis (2014) 20:261 10.3201/eid2002.130522 24447721 PMC3901487

[21] Kracalik IT Blackburn JK Lukhnova L Pazilov Y Hugh-Jones ME Aikimbayev A. Analysing the spatial patterns of livestock anthrax in Kazakhstan in relation to environmental factors: a comparison of local (Gi*) and morphology cluster statistics. Geospat Health (2012) 7:111–26. 10.4081/gh.2012.110 23242686

[22] Kracalik I Abdullayev R Asadov K Ismayilova R Baghirova M Ustun N Changing patterns of human anthrax in Azerbaijan during the post-soviet and preemptive livestock vaccination eras. PLoS Negl Trop Dis (2014) 8:e2985 10.1371/journal.pntd.0002985 25032701 PMC4102439

[23] Cherkasskiy B. A national register of historic and contemporary anthrax foci. J Appl Microbiol (1999) 87:192–5. 10.1046/j.1365-2672.1999.00868.x 10475946

[24] Mullins J Lukhnova L Aikimbayev A Pazilov Y Van Ert M Blackburn JK. Ecological niche modelling of the *Bacillus anthracis* A1. a sub-lineage in Kazakhstan. BMC Ecol (2011) 11:32. 10.1186/1472-6785-11-32 22152056 PMC3260114

[25] Aikembayev AM Lukhnova L Temiraliyeva G Meka-Mechenko T Pazylov Y Zakaryan S Historical distribution and molecular diversity of *Bacillus anthracis*, Kazakhstan. Emerg Infect Dis (2010) 16:789–96. 10.3201/eid1605.091427 20409368 PMC2953997

[26] Joyner T Lukhnova L Pazilov Y Temiralyeva G Hugh-Jones M Aikimbayev A Modeling the potential distribution of *Bacillus anthracis* under multiple climate change scenarios for Kazakhstan. PLoS One (2010) 5:e9596. 10.1371/journal.pone.0009596 20231894 PMC2834750

[27] Kracalik I Lukhnova L Aikimbayev A Pazilov Y Temiralyeva G Blackburn JK. Incorporating retrospective clustering into a prospective cusum methodology for anthrax: evaluating the effects of disease expectation. Spat Spatio-temporal Epidemiol (2011) 2:11–21. 10.1016/j.sste.2010.06.001 22749547

[28] Bezymennyi M Bagamian KH Barro A Skrypnyk A Skrypnyk V Blackburn JK Spatio-temporal patterns of livestock anthrax in Ukraine during the past century (1913–2012). Appl Geogr (2014) 54:129–38. 10.1016/j.apgeog.2014.07.016

[29] Blackburn JK Skrypnyk A Bagamian KH Nikolich M Bezymennyi M Skrypnyk A Anthrax in a backyard domestic dog in Ukraine: a case report. Vector-Borne Zoonotic Dis (2014) 14(8): 615–7. 10.1089/vbz.2013.1519 25072993

[30] Bagamian KH Skrypnyk A Rodina Y Bezymennyi M Nevolko O Skrypnyk V Serological Anthrax Surveillance in wild boar (Sus scrofa) in Ukraine. Vector-Borne Zoonotic Dis (2014) 14:618–20. 10.1089/vbz.2013.1521 25072994

[31] Morgan E Lundervold M Medley G Shaikenov B Torgerson P Milner-Gulland E Assessing risks of disease transmission between wildlife and livestock: the *Saiga* antelope as a case study. Biol Conserv (2006) 131:244–54. 10.1016/j.biocon.2006.04.012

[32] Seleem MN Boyle SM Sriranganathan N. Brucellosis: a re-emerging zoonosis. Vet Microbiol (2010) 140:392–8. 10.1016/j.vetmic.2009.06.021 19604656

[33] Pappas G Papadimitriou P Akritidis N Christou L Tsianos EV. The new global map of human brucellosis. Lancet Infect Dis (2006) 6:91–9. 10.1016/S1473-3099(06)70382-6 16439329

[34] Roth F Zinsstag J Orkhon D Chimed-Ochir G Hutton G Cosivi O Human health benefits from livestock vaccination for brucellosis: case study. Bull World Health Organ (2003) 81:867–76. 14997239 PMC2572379

[35] Gurbanov S Akhmedova S Especially dangerous infections in Azerbaijan. In: Skowroski EW O’Connell KP, editors. Emerging and Endemic Pathogens: Advances in Surveillance, Detection, and Identification. Dordrecht: Springer (2010). p. 39–43.

[36] Abdullayev R Kracalik I Ismayilova R Ustun N Talibzade A Blackburn J. Analyzing the spatial and temporal distribution of human brucellosis in Azerbaijan (1995-2009) using spatial and spatio-temporal statistics. BMC Infect Dis (2012) 12:185. 10.1186/1471-2334-12-185 22873196 PMC3482564

[37] Sanodze L Bautista CT Garuchava N Chubinidze S Tsertsvadze E Broladze M Expansion of brucellosis detection in the country of Georgia by screening household members of cases and neighboring community members. BMC Public Health (2015) 15:459. 10.1186/s12889-015-1761-y 25934639 PMC4432945

[38] Mamisashvili E Kracalik IT Onashvili T Kerdzevadze L Goginashvili K Tigilauri T Seroprevalence of brucellosis in livestock within three endemic regions of the country of Georgia. Prev Vet Med (2013) 110:554–7. 10.1016/j.prevetmed.2012.12.005 23287714

[39] Akhvlediani T Clark DV Chubabria G Zenaishvili O Hepburn MJ. The changing pattern of human brucellosis: clinical manifestations, epidemiology, and treatment outcomes over three decades in Georgia. BMC Infect Dis (2010) 10:346. 10.1186/1471-2334-10-346 21143881 PMC3004911

[40] Gage KL Kosoy MY Natural history of plague: perspectives from more than a century of research*. Annu Rev Entomol (2005) 50:505–28. 10.1146/annurev.ento.50.071803.130337 15471529

[41] Gratz N Rodent reservoirs and flea vectors of natural foci of plague. Plague Man Epidemiol Distrib Surveill Control (1999) 63–96.

[42] Pollitzer R Plague. Geneva: WHO (1954).

[43] Morris LR Blackburn JK Talibzade A Kracalik I Ismaylova R Abdullahyev R Informing surveillance for the lowland plague focus in Azerbaijan using a historic dataset. Appl Geogr (2013) 45:269–79. 10.1016/j.apgeog.2013.09.014

[44] Esmaeili S Azadmanesh K Naddaf SR Rajerison M Carniel E Mostafavi E A serological survey of plague in animals in Western Iran. Emerg. Infect. Dis (2013) 19(9):1549–51. 10.3201/eid1909.121829 PMC3810907 23968721

[45] Staples JE Kubota KA Chalcraft LG Mead PS Petersen JM Epidemiologic and molecular analysis of human tularemia, United States, 1964–2004. Emerg Infect Dis (2006) 12:1113 10.3201/eid1207.051504 16836829 PMC3291054

[46] Charette JD CDC Category explanation (A, B, C) overview. Toxico-Terror Emerg Response Clin Approach Chem Biol Radiol Agents (2007) 755:329.

[47] Hightower J Kracalik IT Vydayko N Goodin D Glass G Blackburn JK. Historical distribution and host-vector diversity of *Francisella tularensis*, the causative agent of tularemia, in Ukraine. Parasit Vectors (2014) 7:1–6. 10.1186/s13071-014-0453-2 25318562 PMC4200231

[48] Golkovskiĭ GM Mitsevich GF Khaĭtovich AB Alekseev EV Korchevskiĭ PG. Natural focus of tularemia on the Kerchen peninsula (Crimea). Zh Mikrobiol Epidemiol Immunobiol (1981) (10):99–101. 7331615

[49] Sjöstedt A Tularemia: history, epidemiology, pathogen physiology, and clinical manifestations. Ann N Y Acad Sci (2007) 1105(1):1–29. 17395726 10.1196/annals.1409.009

[50] Hightower J Examining the Distribution of Francisella Tularensis, the Causative Agent of Tularemia, in Ukraine Using Ecological Niche Modeling. (2012).

[51] Blackburn JK Asher V Stokke S Hunter DL Alexander KA Dances with anthrax: Wolves (*Canis lupus*) kill anthrax bacteremic plains bison (bison bison bison) in Southwestern Montana. J Wildl Dis (2014) 50:393–6. 10.7589/2013-08-204 24484485

[52] Bagamian KH Alexander KA Hadfield TL Blackburn JK. Ante-and postmortem diagnostic techniques for anthrax: rethinking pathogen exposure and the geographic extent of the disease in wildlife. J Wildl Dis (2013) 49:786–801. 10.7589/2013-05-126 24502707

